# How Are Consumers Affected by Taste and Hygiene Ratings When Ordering Food Online? A Behavioral and Event-Related Potential Study

**DOI:** 10.3389/fnins.2022.844027

**Published:** 2022-03-21

**Authors:** Cuicui Wang, Yun Li, Xuan Luo, Huijian Fu, Ziqi Ye, Guangwei Deng

**Affiliations:** ^1^School of Management, Hefei University of Technology, Hefei, China; ^2^Key Laboratory of Process Optimization and Intelligent Decision-Making, Ministry of Education, Hefei University of Technology, Hefei, China; ^3^School of Management, Guangdong University of Technology, Guangzhou, China

**Keywords:** taste rating, hygiene rating, food, cue diagnosticity theory, event-related potentials

## Abstract

With the rapid development of the take-out industry, taste and hygiene ratings as social-based information have been frequently used by online food-ordering platforms to facilitate consumer purchases. The present study aims to uncover the effects of taste and hygiene ratings on online food-ordering decision by incorporating behavioral and neural approaches. The behavioral results showed that a high taste rating induced a higher ordering intention than a low taste rating, and that a high hygiene rating induced a higher ordering intention than a low hygiene rating. The effect of hygiene rating on ordering intention was moderated by taste rating. Hygiene rating had a greater impact on ordering intention when the taste rating was high (vs. low). In addition, inconsistency between taste and hygiene ratings increased the cognitive load and took more time for decision-making. The event-related potential (ERP) data revealed that consumers paid more attention to a high (vs. low) taste rating in the early cognitive process, which was reflected by a larger P2. Subsequently, a more negative N2 was elicited by conflicting ratings than consistent ratings when the taste rating was low. In the relatively late decision-making process, a larger P3 was evoked by consistent than conflicting ratings, suggesting that consumers had more confidence in their decisions for consistent ratings. These findings could help restaurants understand the roles of taste and hygiene rating cues in affecting consumer behavior and prompt those restaurants to adopt effective measures to increase online sales.

## Introduction

With the development of e-commerce, online restaurant ordering, and take-out service have become important parts of daily life. In particular, the outbreak of the COVID-19 pandemic has stimulated the development of the so-called at-home economy since 2020. Consumers could simply browse the information of foods and restaurants at home and order take-outs using mobile food-ordering applications. This process is convenient and efficient for consumers. However, consumers face difficulties in inferring food quality because the foods and restaurants can not be directly observed and physically experienced during online purchase. Since consumer-generated comments and ratings exert informational and social influences on consumer behavior (Utz et al., 2012; Kuan et al., 2014; Tang and Song, 2019), ratings about food taste and hygiene have been increasingly used by the online take-out industry to facilitate consumer decision-making.

Prior research has identified taste and health as important factors that influence food consumption (Vadiveloo et al., 2013). Taste provides information about food quality and is perhaps one of the most important determinants of food preference (Pirastu et al., 2012). Though unable to obtain direct physical experience about taste during online food-ordering, consumers could form taste perception by referring to the reviews posted by other consumers. Hygiene is an important characteristic linked to food safety and consumer health (Kang, 2015). Food manufacturers and restaurants have been trying to brand their healthy image and sell their products through a hygiene perspective (Chandon and Wansink, 2007). The ease of identifying healthful (or hygienic) food has a positive effect on food choice (Liu et al., 2012; Rogers et al., 2016). Not surprisingly, ratings about food taste and hygiene in e-commerce provide crucial information about food quality. Numerous studies have shown that product rating has a notable influence on consumer attitude and can reflect whether the sellers are reliable (Lee et al., 2008). But little empirical research to date has endeavored to understand how taste and hygiene ratings affect consumer choice. It is suggested that the informational social influence of others is highly salient in the context of food consumption (Cruwys et al., 2015). Therefore, we infer that taste and hygiene ratings have positive effects on consumer decision-making during online food-ordering.

According to the cue utilization theory, shopping websites deliver a series of cues which can be divided into intrinsic cues and extrinsic cues, and consumers use both intrinsic and extrinsic cues to assess product quality (Olson and Jacoby, 1972; Dimoka et al., 2012). Intrinsic cues are associated with the direct physical attributes of the product (e.g., ingredients, taste, and smell), whereas extrinsic cues are usually associated with indirect aspects of the product (e.g., product price, brand reputation and online reviews) (Richardson et al., 1994). When consumers are unable to experience intrinsic product cues directly, they tend to make use of extrinsic cues to assess product quality (Wells et al., 2011). Previous research has indicated that extrinsic cues in online stores provide important guidance for consumers’ decisions (Parboteeah et al., 2009; Hu et al., 2010). Since consumers can not directly observe the intrinsic cues of food from mobile food-ordering applications, they are more likely to apply extrinsic cues (i.e., online comments) to assessing food quality. Moreover, building on cue utilization theory, cue-diagnosticity theory suggests that when consumers are faced with multiple extrinsic cues in an online market, they are inclined to prioritize them based on their diagnosticity (Slovic and Lichtenstein, 1971; Skowronski and Carlston, 1987). Diagnosticity refers to the ability of a cue in assisting product evaluation and decision making (e.g., quality assessment and categorization) (Skowronski and Carlston, 1987; Feldman and Lynch, 1988). A more diagnostic cue is given more importance and is more likely to be utilized in product evaluation compared to a less diagnostic cue (Feldman and Lynch, 1988; Purohit and Srivastava, 2001). When multiple cues coexist, the effectiveness of a less diagnostic cue in affecting product evaluation is prone to be enhanced when the more diagnostic cue has a positive valence and inhibited when the more diagnostic cue has a negative valence (Purohit and Srivastava, 2001). For example, Wang et al. (2016) investigated the joint influence of product rating and sales cues on purchase decision and observed that product rating (a more diagnostic cue) had a more pronounced impact on purchasing rate than sales (a less diagnostic cue). When the product rating is high, sales has a positive effect on purchasing rate; but when the product rating is low, sales has no effect on purchasing rate (Wang et al., 2016). In a study examining the joint influence of online rating and product price on purchase decision, it is found that product rating as a more diagnostic cue can positively moderate the effect of price (a less diagnostic cue) on purchase intention (Tang and Song, 2019). However, it is still unknown about the difference of diagnosticity between taste rating and hygiene rating cues. In the present study, taste rating might be perceived with a higher level of diagnosticity compared to hygiene rating given that taste is a key determinant of food quality (Pirastu et al., 2012). It remains to be explored whether these two types of ratings could be interactive in affecting consumer decision-making. According to extant literature on cue diagnosticity, we expect that taste rating would be prioritized in decision making and would moderate the effect of hygiene rating on consumer responses.

Given that neuromarketing approaches are conducive to understanding consumer information processing and decision-making (Yuan et al., 2007; Wang et al., 2016, 2020; Hsu, 2017; Shang et al., 2020), the event-related potential (ERP) method can be used to provide neural evidence for the mechanisms underlying the impact of taste and hygiene rating cues and to understand the priority of information processing between these two factors. Compared with self-report, ERP method can open the black box of the brain and explore the corresponding information processing activities (Dimoka et al., 2012; Kuan et al., 2014). Furthermore, the use of self-reported data is often blamed for bring about subjective biases (Kuan et al., 2014). Therefore, the current study adopted ERP method and behavioral method to examine the underling neural mechanism by considering the effects of two extrinsic rating cues in online food-ordering decision. On the basis of prior studies on information processing and purchase decision-making (Ma et al., 2014; Wang et al., 2016), P2, N2, and P3 were of particular interest to us in the current study.

P2 is a relatively early positive ERP component over frontal regions (Polezzi et al., 2008). It is an attention-associated component that represents early rapid automatic activity, which is followed by the progressive recruitment of slow, elaborative and semantic processing under voluntary control (Correll et al., 2006; Thomas et al., 2007; Ma et al., 2014). Thomas et al. (2007) suggests that P2 reflects the rapid and automatic evaluation of the stimulus in early cognitive stages, and the amplitude of P2 indicates the attention resources invested in the stimulus. The attention resources invested by decision makers are positively correlated with P2 amplitude (Mercado et al., 2006). Therefore, in this study, we speculate that a cue with a high level of diagnosticity (i.e., taste rating) might be prioritized in information processing and grab attention in the relatively early processing stage, which would be indicated by a noticeable P2 component.

N2, another frequently studied ERP component in decision-making research, typically appears after the presence of a stimulus over anterior scalp regions (Folstein and Van Petten, 2008). Unlike P2, N2 belongs to the conscious cognitive processing stages, during which more complex stimuli features could be detected and processed (Yuan et al., 2007). Previous studies have consistently demonstrated that the N2 amplitude is positively associated with conflict detection and cognitive control (Yeung et al., 2004; Yang et al., 2007; Wang et al., 2020). A high degree of conflict induces a more negative N2 amplitude than a low degree of conflict (Yang et al., 2007). For instance, individuals show higher N2 amplitudes when faced with a mismatch between price cues, which suggest the presence of heightened cognitive and decisional conflict (Fu et al., 2019). In the current study, we expect that a low consistency between taste and hygiene ratings would result in greater cognitive conflicts than a high consistency between them.

P3 is a positive ERP component that is maximal over parietal sites and arises at approximately 300–600 ms after stimulus onset (Polich and Kok, 1995). It is associated with conscious evaluation, such as decision difficulty, decision confidence and preference, at the relatively late processing stage of decision making (Nieuwenhuis et al., 2005; Salti et al., 2012). Task-related factors (e.g., task difficulty, task relevance and stimulus probability) have a strong impact on the cognitive processes in decision-making and may result in the variation of P3 amplitude (Jost et al., 2004; Sawaki and Katayama, 2007; Holroyd et al., 2008). In contrast to easy tasks, difficult tasks make individuals more equivocate, decrease their confidence in their judgments, and thus result in a decrease in P3 amplitude (Kok, 2001). Finnigan et al. (2002) demonstrated that the amplitude of P3 was sensitive to decision accuracy and confidence, as a greater P3 amplitude was induced when decision accuracy or confidence was high (vs. low) (Finnigan et al., 2002). Therefore, P3 amplitude reflects task difficulty and decision confidence during decision making (Hillyard et al., 1971). In this study, participants were required to make decisions according to taste and hygiene ratings. We speculate that when taste and hygiene ratings provide consistent predictions, consumers’ confidence in decision making will be enhanced and a larger P3 will be elicited compared to when the ratings provide conflicting predictions.

Taken together, the current study is aimed to reveal how consumers process different types of rating cues in online food-ordering by using behavioral and ERP measures. Taste and hygiene ratings, as two important extrinsic cues, might affect consumers’ perception of the food and the final ordering decision. P2, N2, and P3, three ERP components associated with the evaluation processes, were examined. The findings of this study would contribute to a better understanding of how consumers make online food-ordering decisions when faced with multiple extrinsic cues and help online restaurants to make better use of online ratings to attract potential consumers.

## Study 1: A Behavioral Experiment

In Study 1, we used a behavioral experiment to examine the joint effects of taste rating (high vs. low) and hygiene rating (high vs. low) on online food-ordering decisions. A 2 (taste rating: high vs. low) × 2 (hygiene rating: high vs. low) between-subjects design was employed in Study 1. Therefore, four conditions were created, i.e., high taste rating & high hygiene rating (hereafter HT & HH), high taste rating & low hygiene rating (hereafter HT & LH), low taste rating & high hygiene rating (hereafter LT & HH), and low taste rating & low hygiene rating (hereafter LT & LH).

### Participants

A total of 277 native Chinese (92.4% ranging from 18 to 30 years old, 6.1% ranging from 31 to 40 years old, 1.5% older than 41 years old; 57.4% females) from Hefei University of Technology participated in this experiment online. All participants had online food ordering experience, and they were randomly assigned to one of the four conditions. Specifically, 72 participants were subjected to the HT & HH condition, 69 participants were subjected to the HT & LH condition, 68 participants were subjected to the LT & HH condition, and 68 participants were subjected to the LT & LH condition.

In order to eliminate the confounding effects of personal characteristics (e.g., gender, age, education, and online food ordering frequency), we conducted ANCOVA or chi-square test. The results showed that there were no significant differences of gender (χ^2^(3) = 5.368, *p* > 0.1), age [*F*_(3_,_273)_ = 0.596, *p* > 0.1], education (χ^2^(12) = 9.07, *p* > 0.1), and online food ordering frequency [*F*_(3_,_273)_ = 1.284, *p* > 0.1] among four conditions.

### Experimental Stimuli

In Study 1, we designed a simple smartphone application interface for ordering food online. Four restaurants with different levels of taste and hygiene ratings were created, with restaurant name (using serial numbers), picture (only containing tables and chairs), location (same address for four restaurants), sales (around 500 a month) and restaurant per capita consumption (around 70 Chinese yuan per capita) strictly controlled. Based on the findings of Wang et al. (2016), ratings ranging from 2.00 to 2.25 were classified as low ratings, and ratings ranging from 4.75 to 5.00 were classified as high ratings (1.00 and 5.00 corresponded to the lowest and highest rating scores, respectively). therefore, in the present study, rating scores for the HT & HH condition were set as 4.95 and 4.85, for the HT & LH condition were 4.95 and 2.15, for the LT & HH condition were 2.25 and 4.85, and for the LT & LH condition were 2.05 and 2.15.

### Procedures

The experiment was conducted on wjx.com, which was a professional data collection website in China. Prior to the experiment, participants were informed that the purpose of the research was to understand consumers’ online restaurant ordering behavior. Participants were asked to imagine that “You plan to order take-out through a mobile application. Now you open the food-ordering application and check the information of a restaurant as shown in the following picture.” After reviewing the restaurant information, participants reported how likely they would be to order take-out from this restaurant in a 7-point Likert scale (1 = very unlikely, 7 = very likely). Considering the high internal consistency (α = 0.951), we used the average to form consumer attitude index.

### Results

**FIGURE 1:**
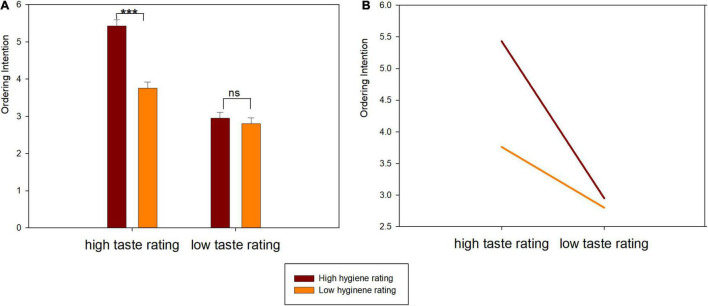
Ordering intention results in Study 1. **(A)** Bar graph. **(B)** Linear graph. ***p < 0.001, ns *p* > 0.05.

A 2 × 2 ANCOVA was conducted with ordering intention as the dependent variable. It revealed significant main effects of taste rating [*F*_(1_,_273)_ = 114.38, *p* < 0.001, η^2^ = 0.30] and hygiene rating [*F*_(1_,_273)_ = 31.76, *p* < 0.001, η^2^ = 0.10]. A high taste rating (*M* = 4.59, S.E. = 0.11) led to a higher ordering intention than a low taste rating (*M* = 2.88, S.E. = 0.12). Similarly, a high hygiene rating (*M* = 4.19, S.E. = 0.11) led to a higher ordering intention than a low hygiene rating (*M* = 3.28, S.E. = 0.11). More importantly, there was a significant interaction effect between taste rating and hygiene rating [*F*_(1_,_273)_ = 22.56, *p* < 0.001, η^2^ = 0.08]. Under the high taste rating condition, participants showed higher ordering intention for a high hygiene rating (HT & HH: *M* = 5.43, S.E. = 0.16) than for a low hygiene rating (HT & LH: *M* = 3.76, S.E. = 0.16; *p* < 0.001). However, under the low taste rating condition, there was no significant difference for ordering intention between a high hygiene rating (LT & HH: *M* = 2.95, S.E. = 0.16) and a low high hygiene rating (LT & LH: M = 2.80, S.E. = 0.16) (*p* > 0.1) (see Figure 1).

### Discussion

The behavioral experiment in Study 1 was conducted to examine the joint effects of taste rating and hygiene rating as two extrinsic cues on consumers’ online food ordering intention. The results revealed that consumers indicated a higher ordering intention from a restaurant with a high taste rating (high hygiene rating) than one with a low taste rating (low hygiene rating), indicating that high ratings for taste or hygiene signaled better restaurant quality and experience. In addition, the effect of hygiene rating on ordering intention was dependent upon taste rating. Hygiene rating had a positive effect on ordering intention when the taste rating was high. whereas hygiene rating did not make a difference when the taste rating was low. According to the cue diagnosticity theory, the effect of a less diagnostic cue on product evaluation is contingent on the valence of the more diagnostic cue when multiple cues coexist (Purohit and Srivastava, 2001; Wang et al., 2016). The findings of Study 1 indicated that taste rating was perceived to be more diagnostic than hygiene rating during online restaurant evaluation and decision making, and thus the effect of the hygiene rating on restaurant online ordering behavior was moderated by taste rating.

Study 1 provided initial evidence for the joint effects of two extrinsic cues on online food ordering intention. However, it remains to be explored about how consumers process the information of taste rating and hygiene rating on the time course and its underlying cognitive mechanism. Therefore, Study 2 investigated the joint effects of taste and hygiene ratings further by examining the brain activities associated with information processing and decision making by employing ERP method.

## Study 2: An Event-Related Potential Experiment

### Participants

Through posting information about the experiment on the campus bulletin board system, we recruited 21 healthy right-handed students (10 females) from Guangdong University of Technology to participate in the experiment. All participants were native Chinese speakers with normal or corrected-to-normal visual acuity and without any history of neurological disorders or mental diseases. They all had experience in ordering food online. The experiment complied with the Declaration of Helsinki and was approved by the Internal Review Board of the Laboratory of Neuromanagement and Decision Neuroscience, Guangdong University of Technology. Before the experiment, all participants provided written informed consent regarding the experiment and the protection of personal privacy, health, safety and dignity. Participants were paid 50 Chinese yuan (approximately 7 USD) after the experiment. The EEG data from two participants were discarded because of excessive recording artifacts (i.e., less than 30 valid trials were remained per condition), leaving valid data from 19 participants (9 females) for the final data analysis. Their age ranged from 19 to 23 years (mean age = 20.5 ± 1.07 years).

### Experimental Stimuli

We collected 200 local restaurant names from popular food-ordering platforms^1^ ^,2^ and allowed the participants to browse them prior to the ERP experiment. A serial number was assigned to each restaurant. To avoid different degrees of familiarity with the restaurants, only the serial number was used to represent each restaurant in the ERP experiment. Moreover, the experiment employed a 2 (taste rating: high vs. low) × 2 (hygiene rating: high vs. low) within-subjects design. That is, the experiment consisted of the same four experimental conditions as Study 1. According to the findings of Wang et al. (2016), ratings ranging from 2.00 to 2.25 were classified as low ratings, and ratings ranging from 4.75 to 5.00 were classified as high ratings. Consequently, five scores were selected from each range to represent high or low ratings in the experiment. The same ten scores were used to manipulate taste and hygiene ratings. The taste and hygiene rating scores were paired randomly, resulting in 100 pairs of ratings. Each pair of ratings was presented twice in the experiment, resulting in 200 trials altogether and 50 trials in each condition. To eliminate the possible confounding effect of reading order, the vertical positions of taste and hygiene ratings were counterbalanced.

### Experimental Procedures

Participants were comfortably seated on a chair in a dimly lit, sound-proof room. The stimuli were presented centrally on a 19-inch computer monitor (1,280 × 1,024 pixels, 60 Hz) against a gray background at a distance of 90 cm in front of each participant. E-Prime 2.0 software (Psychology Software Tools Inc., Pittsburgh, PA, United States) was used to present the stimuli randomly, and a keypad was provided for participants to provide responses.

**FIGURE 2:**
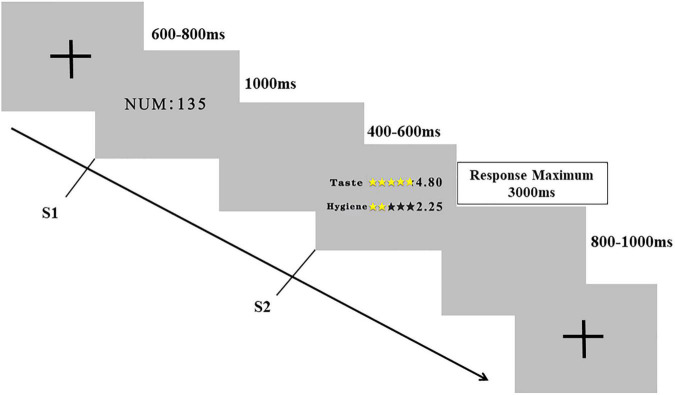
Schematic illustration of one trial in the experimental task of Study 2. Participants were instructed to make a “choose” or “not choose” decision after the presentation of S2 along with taste and hygiene rating information.

Before the ERP experiment started, each participant was given the following introduction: “Imagine that now it is near meal time and you want to order take-out through mobile applications (e.g., Dianping or Meituan). You could see a list of restaurants and check consumer ratings of taste and hygiene about each of the restaurant. You have to decide whether you would like to order food from the restaurant or not after reading the information about the restaurant.” Each participant performed ten practice trials to get familiar with the task. The formal experiment was composed of four blocks, each containing 50 pairs of stimuli. As Figure 2 shows, each trial began with a central fixation cross (+) against a gray background for 600–800 ms, which was followed by a restaurant number (S1) for 1,000 ms. Next, a blank screen was displayed for 400 to 600 ms. After that, a picture with taste and hygiene rating scores (S2) was presented with a duration of 3,000 ms. Participants were asked to decide whether they would like to order food from the restaurant as soon as possible after viewing S2, which would disappear when participants made a response. The response-to-hand assignments were counterbalanced across all participants. Finally, a blank screen was presented for 800 to 1,000 ms before the next trial began. After each block, participants were able to rest for several minutes. The experiment lasted approximately 15 min.

After the ERP experiment, participants were asked to assess the perceived diagnosticity of taste and hygiene ratings on a five-point Likert scale ranging from 1 (very low) to 5 (very high). One item was adapted from Qiu et al. (2012)’s study to measure the perceived diagnosticity of each cue, i.e., to what degree do you think the taste (hygiene) rating is useful for food evaluation and restaurant choice?

### Event-Related Potential Recording and Analysis

The EEG data were recorded with an eego amplifier by using a Waveguard EEG Cap with 64 Ag/AgCl electrodes mounted according to the extended international 10–20 system (both manufactured by ANT Neuro, Enschede, Netherlands). The cephalic (forehead) location in the middle of FPz and Fz served as the ground, and the left mastoid was used as an online reference. Channel data were recorded at a sampling rate of 500 Hz, with online band-pass-filtering from 0.1 to 100 Hz. The electrode impedance was kept below ten kΩ throughout the experiment.

During off-line data analyses, EEG data were analyzed in ASALab 4.10.1 software (ANT Neuro, Enschede, Netherlands). EEG data were re-referenced to the average of the left and the right mastoids off-line. Ocular artifacts were identified and corrected with the eye movement correction algorithm in the ASALab program. The EEG data were digitally filtered with a low-pass filter at 30 Hz (24 dB/octave). For the ERP, the time windows of 200 ms before S2 onset and 800 ms after S2 onset were segmented, with the activity from −200 to 0 ms serving as the baseline. Trials containing amplifier clipping, bursts of electromyography activity or peak-to-peak deflection that exceeded ± 100 mV were excluded from the final averaging. More than 30 sweeps remained in each condition for each participant, which was adequate for achieving stable and reliable measurements of P2, N2, and P3 (Luck, 2005). For each participant, the recorded EEGs over each recording site were grand averaged across each experimental condition. The current experiment examined four conditions varying in taste rating (high vs. low) and hygiene rating (high vs. low).

On the basis of the visual inspection of the grand averaged waveforms and relevant studies on decision-making (Ma et al., 2014; Wang et al., 2016), P2, N2, and P3 components were analyzed. We selected the time windows of 150–190 ms after the onset of S2 for P2, 270–330 ms for N2, and 360–560 ms for P3. According to the brain locations of the ERP components described in the introduction, six electrodes (FC3, FCZ, FC4, C3, CZ, and C4) in the fronto-central and central areas were used for P2 and N2 analyses, and nine electrodes (C3, Cz, C4, CP3, CPz, CP4, P3, Pz, and P4) were used for P3 analysis. Repeated measure analyses of variance (ANOVAs) were conducted separately for P2, N2, and P3. The Greenhouse-Geisser correction was used when necessary (uncorrected df is reported with the ε and corrected *p*-values), and Bonferroni correction was used for multiple paired comparisons. Simple effect analyses were performed when the interaction effect was significant.

### Results

#### Behavioral Data

##### Ordering Rate

**FIGURE 3:**
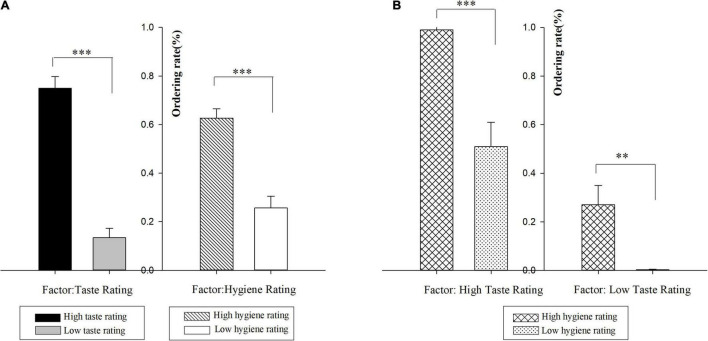
Ordering rate results in Study 2. **(A)** The ordering rates organized according to one factor (taste rating or hygiene rating). **(B)** The ordering rates for all four experimental conditions. The error bars indicate standard error of the mean. ^**^p < 0.01 and ^***^p < 0.001.

Only trials that registered responses in <3 s after S2 onset were included in the behavioral data analyses. Considering the binary decision paradigm in our ERP study, the percentage at which participants decided to order from the restaurant online was called ordering rate, which has similar meaning with ordering intention. The ordering rate and the reaction time (RT) were analyzed separately by repeated-measure ANOVAs with taste rating (high vs. low) and hygiene rating (high vs. low) as within-subject factors. It showed significant main effects of taste rating [*F*_(1_,_18)_ = 78.77, *p* < 0.001, η^2^ = 0.81] and hygiene rating [*F*_(1_,_18)_ = 28.12, *p* < 0.001, η^2^ = 0.61] on ordering rate (see Figure 3A). A high taste rating (*M* = 0.75, S.E. = 0.05) led to a higher ordering rate than a low taste rating (*M* = 0.13, S.E. = 0.04). Similarly, a high hygiene rating (*M* = 0.63, S.E. = 0.04) led to a higher ordering rate than a low hygiene rating (*M* = 0.26, S.E. = 0.05). Furthermore, the differentiated rate (the difference of ordering rate between high and low rating conditions) of the taste rating (*M* = 0.62, S.E. = 0.07) was marginally larger than that of the hygiene rating (*M* = 0.37, S.E. = 0.07) (*t*_(18)_ = 1.77, *p* < 0.1). Interestingly, a marginally significant interaction effect was observed between taste and hygiene ratings [*F*_(1_,_18)_ = 4.09, *p* < 0.1, η^2^ = 0.19] (see Figure 3B). Simple effect analyses showed that under the high taste rating condition, the ordering rate for a high hygiene rating (HT & HH: *M* = 0.99, S.E. = 0.05) was higher than that for a low hygiene rating (HT & LH: *M* = 0.51, S.E. = 0.10; *p* < 0.001). Under the low taste rating condition, the ordering rate for a high hygiene rating (LT & HH: *M* = 0.27, S.E. = 0.08) was also higher than that for low hygiene rating (LT & LH: *M* = 0.003, S.E. = 0.002) (*p* < 0.01). But the difference between high and low hygiene ratings was marginally larger when the taste rating was high (vs. low) (0.48 vs. 0.27, *p* < 0.1).

##### Reaction Time

**FIGURE 4:**
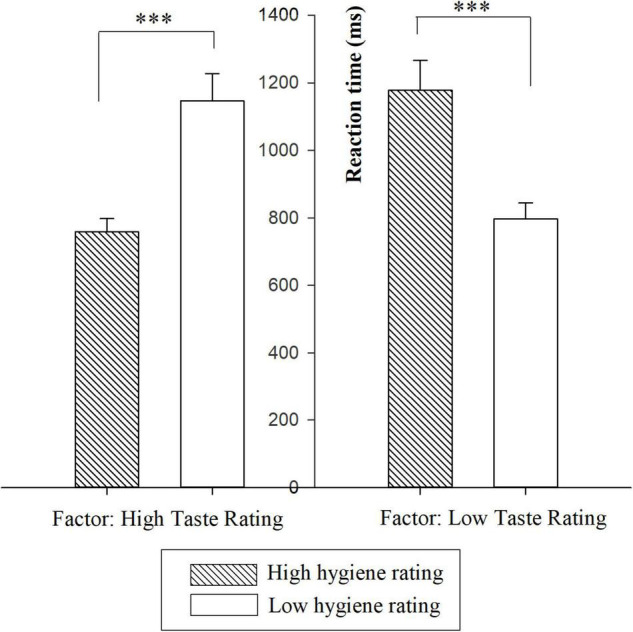
Reaction time (RT) results for all experimental conditions in Study 2. The error bars indicate standard error of the mean. ^***^p < 0.001.

All RTs were longer than 300 ms, therefore no extremely fast RTs were found and included in the analysis. The ANOVA showed insignificant main effects of taste and hygiene ratings, but a significant interaction effect between them [*F*_(1_,_18)_ = 45.63, *p* < 0.05, η^2^ = 0.70]. Simple contrasts showed that under the high taste rating condition, the RT for a high hygiene rating (HT & HH: *M* = 758.68, S.E. = 39.23) was significantly shorter than that for a low hygiene rating (HT & LH: *M* = 1144.54, S.E. = 80.67) (*p* < 0.001). Under the low taste rating condition, the RT for a high hygiene rating (LT & HH: *M* = 1178.40, S.E. = 88.24) was significantly longer than that for a low hygiene rating (LT & LH: *M* = 797.31, S.E. = 46.80) (*p* < 0.001) (Figure 4).

##### Perceived Diagnosticity

A paired *t*-test was performed to compare the perceived diagnosticity of different types of ratings. It demonstrated that the perceived diagnosticity of taste rating (*M* = 4.16, S.E. = 0.21) was significantly higher than that of hygiene rating (*M* = 3.32, S.E. = 0.24; *t*_(18)_ = 2.109, *p* < 0.05).

#### Event-Related Potential Data

**FIGURE 5:**
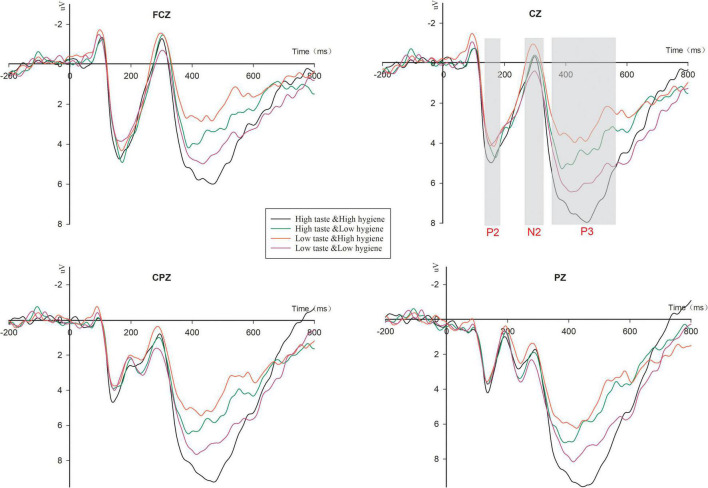
Event-related potential (ERP) results in Study 2. Grand-average ERP waveforms of the frontal, fronto-central, and central regions recorded from the FCz, Cz, CPz, and Pz electrodes.

The stimulus-locked grand-average ERP across four conditions at four representative midline electrodes are shown in Figure 5.

Three-way 2 (taste rating: high vs. low) × 2 (hygiene rating: high vs. low) × 6 (electrodes: FC3, FCZ, FC4, C3, CZ, and C4) within-subject ANOVA for P2 in the time window from 150 to 190 ms was conducted. A significant main effect of taste rating [*F*_(1_,_18)_ = 7.02, *p* < 0.05, η^2^ = 0.28] was observed, as a high taste rating evoked a larger P2 (*M* = 4.05, S.E. = 0.43) than a low taste rating (*M* = 3.51, S.E. = 0.47). But the main effect of hygiene rating and the interaction between taste and hygiene ratings were not significant.

To further explore the informational conflict effects of taste and hygiene ratings, we conducted an ANOVA on N2 amplitude between 270 and 330 ms. It revealed insignificant main effects of taste and hygiene ratings, but a significant interaction effect between taste and hygiene ratings [*F*_(1_,_18)_ = 4.88, *p* < 0.05, η^2^ = 0.21]. When the taste rating was high, the amplitude of N2 did not differ between a high hygiene rating (HT & HH: *M* = 0.52, S.E. = 0.50) and a low hygiene rating (HT & LH: *M* = 1.2, S.E. = 0.63; *p* > 0.1). However, when the taste rating was low, the amplitude of N2 for a high hygiene rating (LT & HH: *M* = −0.04, S.E. = 0.61) was marginally larger than that for a low hygiene rating (LT & LH: *M* = 0.69, S.E. = 0.67; *p* < 0.1). We also conducted a 2 (rating consistency: consistent vs. conflicting) × 6 (electrodes: FC3, FCZ, FC4, C3, CZ and C4) within-subject repeated ANOVA for N2 amplitude. When the taste and hygiene ratings gave consistent predictions, the situation was defined as a consistent condition (including HT & HH and LT & LH); otherwise, it was defined as a conflicting condition (including HT & LH and LT & HH). The results showed a significant main effect of rating consistency [*F*_(1_,_18)_ = 4.89, *p* < 0.05, η^2^ = 0.21], as the conflicting condition (*M* = 0.07, S.E. = 1.17) elicited a more negative N2 than the consistent condition (*M* = 1.21, S.E. = 1.07).

Regarding the P3 component, an ANOVA was performed on P3 amplidute in the time window from 360 to 560 ms. The results revealed a significant main effect of taste rating [*F*_(1_,_18)_ = 8.37, *p* = 0.010, η^2^ = 0.317], but no salient main effect of hygiene rating. The P3 amplitude for a high taste rating (*M* = 6.16, S.E. = 0.730) was larger than that for a low taste rating (*M* = 5.22, S.E. = 0.66). In addition, a significant interaction effect was observed between taste and hygiene ratings [*F*_(1_,_18)_ = 48.38, *p* < 0.001, η^2^ = 0.73]. Simple effect analyses showed that when the taste rating was high, a high hygiene rating (HT & HH: *M* = 7.53, S.E. = 0.85) evoked a greater P3 amplitude than a low hygiene rating (HT & LH: *M* = 4.80, S.E. = 0.67, *p* < 0.001); but when the taste rating was low, a high hygiene rating (LT & HH: *M* = 4.20, S.E. = 0.57, *p* = 0.000) evoked a smaller P3 amplitude than a low hygiene rating (LT & LH: *M* = 6.23, S.E. = 0.82, *p* = 0.010). In other words, the consistent ratings evoked greater P3 amplitudes than the conflicting ratingss (i.e., HT & HH > HT & LH, LT & LH > LT & HH). We also conducted a 2 (rating consistency: consistent vs. conflicting) × 9 (electrodes: C3, Cz, C4, CP3, CPz, CP4, P3, Pz, and P4) within-subject repeated ANOVA for P3 amplitude. The results showed a significant main effect of rating consistency [*F*_(1_,_18)_ = 48.38, *p* < 0.001, η^2^ = 0.73]. The consistent condition (*M* = 6.88, S.E. = 0.79) elicited a more positive amplitude of P3 than the conflicting condition (*M* = 4.498, S.E. = 0.591).

### Discussion

The ERP experiment of Study 2 was conducted to reveal the brain activities associated with information processing of taste rating and hygiene rating when ordering food online. Behaviorally, the ordering rate and RT were examined. Neurally, the ERP components of P2, N2 and P3 were examined, which provided neural evidence for understanding how taste and hygiene ratings affect consumer responses in the brain.

At the behavioral level, a high taste rating (high hygiene rating) induced a higher ordering rate than a low taste rating (low hygiene rating). According to cue utilization theory, our findings indicate that high ratings for both taste and hygiene dimensions signal better restaurant quality than low ratings. Furthermore, the differentiated rate (the difference of ordering rate between a high rating and a low rating) of the taste rating was marginally greater than that of the hygiene rating, indicating that a change in taste rating had a larger impact on consumer behavior than that in hygiene rating. More importantly, we found a marginally significant interaction between taste and hygiene ratings on ordering rate. The difference between high and low hygiene ratings was marginally larger when the taste rating was high (vs. low), suggesting that hygiene rating had a greater impact on ordering rating under the high taste rating condition.

In terms of RT, the HT & LH condition led to a longer RT than the HT & HH condition, and the LT & HH condition led to a longer RT than the LT & LH condition. Prior studies have shown that task completion time is associated with task difficulty and cognitive load, and the greater the task difficulty, the higher the cognitive load perceived by participants (Leuthold et al., 2011; Wang et al., 2016; Jin et al., 2017). It generally requires a longer time for more in-depth cognitive processing. In the current study, longer RTs were observed when the two types of ratings provided conflicting (vs. consistent) predictions. In line with prior studies, the longer RTs for the conflicting (vs. consistent) conditions reveals a greater level of cognitive load experienced in decision-making.

At the brain level, three ERP components, P2, N2, and P3, were identified in this study. A greater P2 amplitude was observed for the high taste rating condition than for the low taste rating condition. P2 is an early ERP component that is associated with the attention resources invested in the stimulus, and a larger P2 amplitude could be induced when more attention was allocated to the stimulus in the automatic evaluation process (Mercado et al., 2006; Thomas et al., 2007; Ma et al., 2014). In this study, participants might automatically evaluate taste rating in the relatively early stage of information processing and devote more attention resources to a high taste rating than a low taste rating. However, no clear difference in P2 amplitude was found between high and low hygiene ratings. According to cue-diagnosticity theory, we speculate that taste rating might be processed with priority during early cognitive processing since it is more diagnostic of food quality than hygiene rating. The finding of P2 was in consonance with the behavioral result, which demonstrated that taste rating was more impactful than hygiene rating in consumer decisions.

Following P2, a more negative N2 component was observed for conflicting ratings (i.e., a combination of HT & LH and LT & HH) than consistent ratings (i.e., a combination of HT & HH and LT & LH). N2 is positively associated with conflict detection and cognitive control (Yang et al., 2007; Forster et al., 2011; Spapé et al., 2011). The larger N2 for the conflicting ratings suggests a stage of informational conflict detection when consumers encountered extrinsic cues with inconsistent predictions. In other words, conflicting ratings induced a higher level of cognitive conflict and required the exertion of more cognitive control than consistent ratings. Furthermore, the salience of the conflict was contingent upon taste rating. When the taste rating was low, a high hygiene rating (conflicting condition) evoked a more negative N2 amplitude than a low hygiene rating (consistent condition). However, when the taste rating was high, no significant difference was found in N2 amplitude between a high hygiene rating (consistent condition) and a low hygiene rating (conflicting condition). Given that a high taste rating captures more attention resource than a low taste rating, as reflected by P2, less attention might be paid to hygiene rating when the taste rating was high. Hence the difference between the HT & HH and HT & LH conditions might be overlooked in this cognitive stage. In contrast, when the taste rating was low, more attention could be allocated to hygiene rating. Hence the conflict between taste and hygiene ratings could be well detected, resulting in a larger N2 for the LT & HH (vs. LT & LH) condition.

Regarding P3 component, this study revealed a greater P3 amplitude for the high (vs. Low) taste rating condition. P3 has been associated with task difficulty and individuals’ confidence in decision-making (Nieuwenhuis et al., 2005; Salti et al., 2012). When an individual has a high (vs. low) degree of confidence in judgment or decision-making tasks, a greater P3 amplitude will be evoked (Finnigan et al., 2002). Thus, the larger P3 for the high (vs. low) taste rating might indicate that participants had a higher degree of confidence when the restaurant has a high taste rating. More importantly, the significant interaction between taste and hygiene ratings showed that consistent ratings triggered larger P3 amplitudes than conflicting ratings (i.e., HT & HH > HT & LH, and LT & LH > LT & HH). These findings suggest that consumers encounter less difficulty and are more confident in their decisions when consistent (vs. conflicting) ratings are presented to them. The RT results also provide support for the notion that LPP is representative of decision difficulty and/or decision confidence, as a shorter time was required to make a decision when the ratings provided consistent (vs. conflicting) predictions (i.e., HT & HH < HT & LH, and LT & LH < LT & HH).

## General Discussion

### Key Findings

This study investigated the joint effects of two extrinsic cues, i.e., taste and hygiene ratings, on online food-ordering decisions. By incorporating behavioral and ERP approaches, we found taste and hygiene ratings are weighed differently in online food-ordering decisions, and we also uncovered the brain activities associated with information processing and decision making.

First, a high taste rating (high hygiene rating) had more positive influence on online food-ordering decision than a low taste rating (low hygiene rating). More importantly, taste rating is perceived to be more diagnostic than hygiene rating during online food-ordering decision making, and the effect of hygiene rating on online food purchase behavior is moderated by taste rating. The behavioral data collected by Study 1 and Study 2 provided consistent supports for the above conclusion. The results were not entirely consistent with the findings of Hu et al. (2010) and Miyazaki et al. (2005), which suggested that when extrinsic cues were inconsistent, negative, but not positive, cues tended to dominated consumer evaluation (Miyazaki et al., 2005; Hu et al., 2010). In the current study, the effect of high (positive) or low (negative) hygiene rating cues was modulated by the valence of taste rating. The findings of the present study are concordant with cue dianosticity theory, which suggests that the effect of a less diagnostic cue on product evaluation is contingent on the valence of the more diagnostic cue when multiple cues coexist (Purohit and Srivastava, 2001; Wang et al., 2016). Take Purohit and Srivastava (2001)’s study as an example, they reported that product warranty (a less diagnostic cue) had a positive impact on consumer response when brand reputation (a more diagnostic cue) was high and had no effect when brand reputation was low. Furthermore, in a study examining the joint influence of online rating (a more diagnostic cue) and product price (a less diagnostic cue) on purchase decision, it is found that product rating positively moderates the effect of price on purchase intention (Tang and Song, 2019). In Study 2, the self-reports collected after the ERP experiment confirms that taste rating is perceived to be more diagnostic than hygiene rating during product evaluation and decision making. Therefore, it’s not surprising that taste rating has a larger impact on ordering rate than hygiene rating, and the effect of the hygiene rating on ordering rate is moderated by taste rating.

Second, taste rating is weighed more heavily than hygiene rating in consumer information processing and decision making, which is supported by ERP data collected from Study 2. The ERP component of P2 was more sensitive to taste rating than hygiene rating in the relatively early automatic cognitive stage, suggesting that taste rating is prioritized during information processing. The effect of hygiene rating on conflict-related N2 component was dependent upon taste rating, as the difference between high and low hygiene ratings was only present when the taste rating was low (vs. high). Moreover, a main effect of taste rating on P3 component was observed instead of hygiene rating. Consumers are more confident in their decisions when the taste rating is high (vs. low). In line with the cue-diagnosticity theory, we suggest that taste rating is more diagnostic and is weighed more heavily than hygiene rating in consumer information processing and decision making.

### Implication for Practice

The rapid advance in electronic commerce as well as the ongoing COVID-19 pandemic have made online food-ordering increasingly prevalent. The extrinsic cues provided by online food-ordering platforms reduce the asymmetry of information between sellers and consumers and largely facilitate consumer decision-making. By addressing how two types of extrinsic cues (i.e., taste and hygiene ratings) affect online food-ordering decision, the findings of the present study may be of great interest to online food-ordering platforms and restaurants. First, they should realize the importance of boosting taste and hygiene ratings, since both ratings positively predict ordering rate. E-sellers of take-out service should take advantage of these two extrinsic rating cues to exert positive social influence. Second, taste rating should be enhanced with priority when the resources that a restaurant could utilize are limited. Taste rating has a larger impact on consumer responses and the effect of hygiene rating on consumer responses is dependent upon taste rating. Therefore, when online marketing resources are limited for restaurants, they should pay more attention to improving the taste or texture and innovating flavors of food in the first place, with the aim of improving the taste rating. Last but not least, it’s recommended that consistent positive ratings are provided to the consumers, as consistent ratings lower the difficulty of decision making and increase consumers’ confidence in their decisions. In a fast-paced era of mobile internet, online sellers have to strive to make it easier for consumers to make a fast and right decision.

### Limitations and Future Research

This work has some limitations that should be acknowledged. First, the online food-ordering decision scenario in the study 2 was simplified compared to real life decision making, because ERP experiments must follow strict requirements for environment, equipment and materials. Second, most participants were college students. Examining participants with different demographic backgrounds would provide a more comprehensive and generalized understanding of the brain activities of general consumers in the decision-making process in online ordering and a greater sample size may increase the robustness of the current results. Third, this work only considered two types of ratings (i.e., taste and hygiene ratings) on online food-ordering platforms, leaving opportunities for investigating other types of ratings (e.g., ratings about food appearance and delivery service) and other characteristics of online reviews (e.g., reviewer reputation and expertise) in future research.

## Data Availability Statement

The raw data supporting the conclusions of this article will be made available by the authors, without undue reservation.

## Ethics Statement

The studies involving human participants were reviewed and approved by the Internal Review Board of the Laboratory of Neuromanagement and Decision Neuroscience, Guangdong University of Technology. The patients/participants provided their written informed consent to participate in this study.

## Author Contributions

CW, YL, and HF conceived and designed the two experiments and performed the ERP experiment. YL, ZY, and GD performed the behavioral experiment. YL, XL, and ZY analyzed the data. CW, YL, XL, and HF wrote and refined the article. All authors contributed to the article and approved the submitted version.

## Conflict of Interest

The authors declare that the research was conducted in the absence of any commercial or financial relationships that could be construed as a potential conflict of interest.

## Publisher’s Note

All claims expressed in this article are solely those of the authors and do not necessarily represent those of their affiliated organizations, or those of the publisher, the editors and the reviewers. Any product that may be evaluated in this article, or claim that may be made by its manufacturer, is not guaranteed or endorsed by the publisher.

## Footnotes

1
www.Dianping.com

2
www.Meituan.com
